# Air Pollution Characteristics and Health Risks in the Yangtze River Economic Belt, China during Winter

**DOI:** 10.3390/ijerph17249172

**Published:** 2020-12-08

**Authors:** Mao Mao, Haofei Sun, Xiaolin Zhang

**Affiliations:** 1 School of Binjiang, Nanjing University of Information Science & Technology, Wuxi 214105, China; 2 Key Laboratory for Aerosol-Cloud-Precipitation of China Meteorological Administration, School of Atmospheric Physics, Nanjing University of Information Science & Technology, Nanjing 210044, China; sunhaofei20@mails.ucas.ac.cn

**Keywords:** particulate matter, health risks, major pollutant, HAQI, Yangtze River Economic Belt

## Abstract

The air pollution characteristics of six ambient criteria pollutants, including particulate matter (PM) and trace gases, in 29 typical cities across the Yangtze River Economic Belt (YREB) from December 2017 to February 2018 are analyzed. The overall average mass concentrations of PM_2.5_, PM_10_, SO_2_, CO, NO_2_, and O_3_ are 73, 104, 16, 1100, 47, and 62 µg/m^3^, respectively. PM_2.5_, PM_10_, and NO_2_ are the dominant major pollutants to poor air quality, with nearly 83%, 86%, and 59%, exceeding the Chinese Ambient Air Quality Standard Grade I. The situation of PM pollution in the middle and lower reaches is more serious than that in the upper reaches, and the north bank is more severe than the south bank of the Yangtze River. Strong positive spatial correlations for PM concentrations between city pairs within 300 km is frequently observed. NO_2_ pollution is primarily concentrated in the Suzhou-Wuxi-Changzhou urban agglomeration and surrounding areas. The health risks are assessed by the comparison of the classification of air pollution levels with three approaches: air quality index (AQI), aggregate AQI (AAQI), and health risk-based AQI (HAQI). When the AQI values escalate, the air pollution classifications based on the AAQI and HAQI values become more serious. The HAQI approach can better report the comprehensive health effects from multipollutant air pollution. The population-weighted HAQI data in the winter exhibit that 50%, 70%, and 80% of the population in the upstream, midstream, and downstream of the YREB are exposed to polluted air (HAQI > 100). The current air pollution status in YREB needs more effective efforts to improve the air quality.

## 1. Introduction

In the past decade, air pollution levels over China have been far higher than the World Health Organization (WHO) guidelines, with unprecedented urbanization and economic development [1,2,3,4]. The coexistence of particulate matter (PM) and gaseous pollutants (e.g., SO_2_ and NO_x_) from urban traffic and regional industrial sources can interfere with the chemical composition, optical properties, and mixing state of aerosol particles [5]. In recent years, through the adoption of a series of control measures, the air quality has gradually improved, and the number of sunny days has been continuously increasing [6,7]. However, urban fog and haze weather is still a major threat to human health, as well as the environment [8]. In early 2013, the Ministry of Environmental Protection (MEP) of China began to release real-time urban air quality data. The concentrations of six criteria pollutants, especially PM_2.5_, NO_2_, and O_3_, are frequently above the Chinese Ambient Air Quality Standard (CAAQS) [9]. The pollutants have caused the strong concern of the public, because long-term exposure to air pollution potentially poses adverse effects on human health, triggers cardiovascular and respiratory diseases, and causes damage to human immune systems [10,11,12,13,14].

The Yangtze River Economic Belt (YREB) along the Yangtze River, with Shanghai as the leader, is one of the most important areas for China’s economic growth. In 2016, “the outline of the YREB Development Plan” officially became a national development strategy, aiming to push forward the new YREB urbanization [15]. The long-term high-intensity energy consumption and explosive growth of automobile traffic have caused most cities in the developed YREB to face more severe air pollution problems than other cities in China [16]. It is of great significance to analyze the spatial distribution characteristics of air pollutants for taking effective measures to control and reduce pollution. Previous research on air pollution distribution patterns mainly focus on a few key cities or urban agglomerations over the YREB. Zhao et al. explored the spatiotemporal variations of six criteria air pollutants and influencing factors (sources and meteorology conditions) in the city clusters of the Sichuan Basin, which are located on the upper reaches of the YREB, and the results indicated that both PM_2.5_ and O_3_ were simultaneously high in the West Sichuan Basin while PM_2.5_ in the South Sichuan Basin was much higher than that in other regions [17]. Numerous joint studies on particulate and gaseous pollutants have been carried out in representative urban cities in the Yangtze River Delta region, located on the down reaches of the YREB, such as Shanghai and Nanjing [18,19,20,21]. In our earlier study, concentrating on three metropolises in the YREB, we checked the current air pollution status in Chongqing, Wuhan, and Nanjing and compared the results with earlier peer-reviewed studies [22]. The lately published available air quality observation data of six pollutants from more cities over the YREB make it possible to look into the spatial distributions of the YREB. Additionally, when evaluating the impacts of pollutant on human health, the air quality index (AQI) method on account of the maximum concentration of six pollutants was frequently adopted, but the single pollutant index was more impossible to reflect the actual air pollution level comprehensively [23,24,25]. Subsequently, to solve the defect of the AQI, the aggregate AQI (AAQI) and health risk-based AQI (HAQI) were proposed by Kyrkilis et al. and Wong et al., with considering the comprehensive health effects of exposure to multi-pollutants, respectively [23,24]. Some studies compared the three approaches in several major megacities in China and pointed out that the AQI system clearly underestimated the combined impact of multiple pollutants on health risks [25,26,27,28]. The YREB is affected by high concentrations of air pollutants, so the health risks associated with exposure to multi-pollutants might be more unique.

To the best of the authors’ knowledge, the systematic analyzation of air pollution characteristics, including particulate, gaseous pollutants, and corresponding human health risks in the overall YREB, are still lacking so far. To fill up the research gap, the major objectives of this work are organized as follows: (1) to reveal the spatial characteristics of six criteria pollutants (PM_2.5_, PM_10_, SO_2_, CO, NO_2_, and O_3_); (2) to investigate the regional relationship of PM pollution between different city pairs; (3) to identify the proportion of major pollution in the upstream, midstream, and downstream of the YREB; and (4) to assess the health effects of air pollutants on people and identify the proportion of humans exposed to polluted air based on the comparative analysis of three approaches: AQI, AAQI, and HAQI during the period 1 December 2017 to 28 February 2018 in 29 typical cities over the YREB. The findings will be helpful to understand the current situation of environmental quality and provide a reference for air pollution alleviation in YREB if necessarily in time.

## 2. Data and Methodology

### 2.1. Study Region

**Figure 1:**
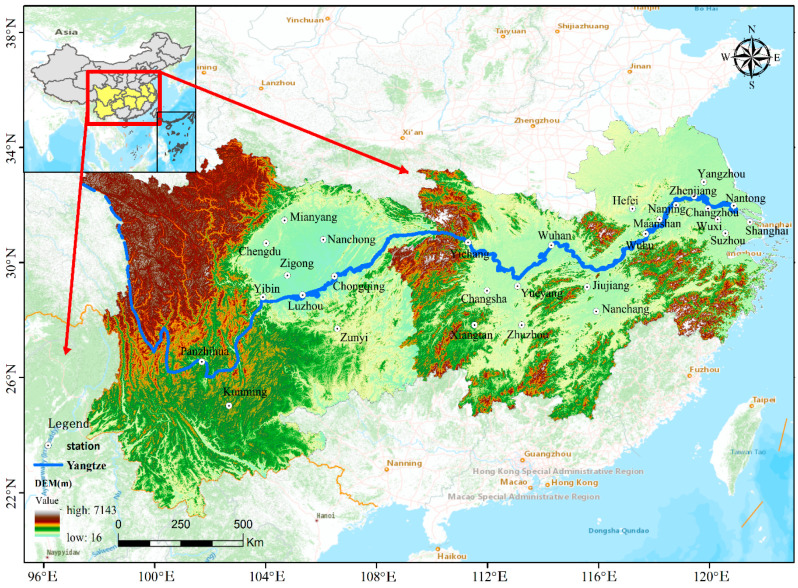
Location of the 29 cities in the Yangtze River Economic Belt (YREB) selected in this study. Yichang and Jiujiang are the boundary points between the upstream, midstream, and downstream of the YREB.

The YREB encompasses nine provinces (Yunnan, Sichuan, Guizhou, Hunan, Hubei, Jiangxi, Anhui, Jiangsu, and Zhejiang) and two municipalities (Chongqing and Shanghai) and stretches across China from the east to west. Its geographical area is about 2.05 million km^2^, accounting for 21.4% of the whole country’s land mass. The YREB accounts for more than 40% of the national total population, as well as gross domestic product (GDP), which makes it the most populous and prosperous river economic belt in China. The YREB is an inland river economic zone with global influence. Under the implementation of various policies and guidelines, the economic growth space has expanded from coastal areas to inland areas along the Yangtze River quickly; however, the efficient environmental pollution control across provinces is still not perfected. On the basis of the geographical location, the YREB is divided into three major areas (the upstream, the midstream, and the downstream) to better understand the regional air pollution characteristics and associated health risks. The detail data of 29 typical cities selected in this study are listed in Table 1, and the spatial distribution of each city is illustrated in Figure 1.

### 2.2. Date Source

In this study, the ground-based hourly mass concentrations of PM_2.5_, PM_10_, SO_2_, CO, NO_2_, and O_3_—8 h from 1 December 2017 to 28 February 2018—were collected at each site from the official website of the China National Environmental Monitoring Centre [29]. The 8-h, daily, and wintertime concentrations of the six pollutants were obtained by averaging the hourly data of all monitoring stations in each city. The diurnal concentrations of each pollutant were calculated only when there were more than 16 h of valid data. Description of the quality assurance and controls were reported in the previous literature [22]. The basic information, such as GDP, population, and vehicle counts of studied cities, was downloaded from the Chinese Statistics Web Links [30].

### 2.3. Methods

#### 2.3.1. AQI

The AQI calculation approach refers to the CAAQS [31], which firstly calculates the individual air quality index (AQI*_i_*) for each pollutant based on the 24-h average concentrations of six pollutants and then chooses the maximum AQI*_i_* as the overall AQI (see Supporting Information) [32].

#### 2.3.2. AAQI

The *AAQI*, considering the combined effects of the six criteria pollutants, is calculated through Formula (1) [23,33]:

(1) $$AAQI={\left({\sum_{i=1}^{n}\left(AQI_{i}\right)}^{\rho }\right)}^{\frac{1}{\rho }}$$

where *ρ* is an empirical constant. Similar to other literatures, *ρ* = 2 was adopted in this paper [25,26,27].

#### 2.3.3. HAQI

To explicate the established exposure–response relationship between various atmosphere contaminants and health risks, the concept of total excess risk (*ER_total_*) of exposure to six pollutants was proposed [34]. First, the relative risk (*RR_i_*) of a pollutant *i* was calculated by Formula (2):

(2) $$RR=\exp\left[\beta_{i}\left(C_{i}-C_{i}{}_{,0}\right)\right],C_{i}>C_{i}{}_{,0}$$

where *C_i_* represents the measured concentration of pollutant *i*, and *C_i,0_* represents the threshold concentration of pollutant *i* (the upper risk limit of CAAQS daily Grade II in this study). The *β* (0.038% per μg/m^3^ for PM_2.5_, 0.032% per μg/m^3^ for PM_10_, 0.081% per μg/m^3^ for SO_2_, 0.13% per μg/m^3^ for NO_2_, 0.048% per μg/m^3^ for O_3_, and 3.7% per mg/m^3^ for CO [2]) is the exposure–response coefficient and represents the excess health risk caused by the increase in per-unit pollutant *i* when *C_i_* > *C_i,0_*. There is no excess health risk for pollutant *i* as *C_i_* £ *C_i,0_* (*RR_i_* = 1).

The excess risk of each pollutant *i* (*E*R*_i_*) and the *ER_total_* of six pollutants are calculated by Formula (3):

(3) $$ER_{total}=\sum_{i=1}^{n}ER_{i}=\sum_{i=1}^{n}\left(RR_{i}-1\right)$$

Supposing the *ER* of a pollutant *i* is equal to *ER_total_*, its equivalent relative risk (RR^*^) and equivalent pollutant concentration (C_i_^*^) can be derived as Formulas (4) and (5) [25]:

(4) $$RR^{*}=ER_{total}+1=\exp\left[\beta_{i}(C_{i}^{*}-C_{i,0})\right]$$

(5) $$C_{i}^{*}=\frac{\ln(RR^{*})}{\beta_{i}}+C_{i,0}$$

C_i_^*^ is the aggregate risk of all pollutants; hence, the *HAQI_i_* can be obtained by Formula (6) [25]:

(6) $$\begin{array}{l} HAQI_{i}=\frac{AQI_{i,j}-AQI_{i,j-1}}{C_{i,j}-C_{i,j-1}}\times (C_{i}^{*}-C_{i,j}{}_{-1})+AQI_{i,j-1},j>1\\ HAQI_{i}=AQI_{i,1}\frac{C_{i}^{*}}{C_{i,1}},j=1 \end{array}$$

where *C_i,j_* and *C_i,j−_*_1_ are the nearby high and low values of C_i_^*^, and *AQI_i,j_* and *AQI_i,j−1_* are the individual air quality indexes for *C_i,j_* and *C_i,j−_*_1_ (Supplementary Table S1), respectively.

After getting each *HAQI_i_*, the overall *HAQI* is then calculated by choosing the maximum *HAQI_i_* of the six pollutants as Formula (7):

(7) $$HAQI=\max(HAQI_{1},HAQI_{2},HAQI_{3},HAQI_{4},HAQI_{5},HAQI_{6})$$

## 3. Results and Discussion

### 3.1. Wintertime Air Quality Overview

**Figure 2:**
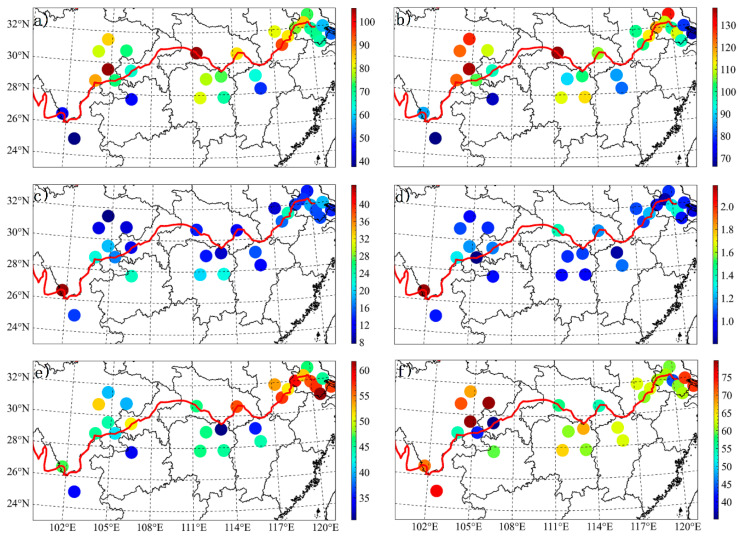
Wintertime average concentrations of particle matter (PM)_2.5_ (**a**), PM_10_ (**b**), SO_2_ (**c**), CO (**d**), NO_2_ (**e**), and 8-h peak O_3_ (**f**). The unit is μg/m^3^ for PM_2.5_, PM_10_, SO_2_, NO_2_, 8-h peak O_3_, and mg/m^3^ for the CO.

Figure 2 illustrates the spatial variations of the averaged concentrations of the six criteria air pollutants for 29 typical cities across the YREB during the entire study time period (1 December 2017–28 February 2018). For PM_2.5_ pollution, conspicuous spatial heterogeneity is observed. The mass concentrations are high, with an increasing spatial pattern from upstream (~69 µg/m^3^) to midstream (~76 µg/m^3^) and downstream (~74 µg/m^3^). Regions with serious PM_2.5_ pollution and relatively clean air quality are roughly diagonally distributed, and the former is mainly located on the north bank of the middle and lower reaches (~80 µg/m^3^), while the latter is primarily concentrated on the south bank of the upper reaches (~45 µg/m^3^) of the Yangtze River. In particular, the PM_2.5_ mass loadings in the Sichuan Basin urban agglomeration are also high. In contrast to the summer, spring, and autumn concentrations, the wintertime concentrations are generally higher due to meteorological conditions unfavorable to pollutant diffusion (inversion, lower boundary layer, etc.) and the increased energy consumption caused by anthropogenic activities (coal, fossil oil, etc.) [35,36,37,38,39]. PM_10_ displays similar spatial distributions, and its concentrations range from 80 to 130 µg/m^3^ for the majority of cities (Table 2). Especially, Zigong in Sichuan Province and Yichang in Hubei Province are the two heavily polluted cities and have the highest PM_2.5_ (PM_10_) levels of all cities in the winter, up to ~105 (137) µg/m^3^.

A smaller difference is observed for both CO and SO_2_ in all cities. The only exception is Panzhihua, located within an important metallogenic belt in Southwest China. The sulfur-containing minerals and coal-fired heating directly lead to high concentrated CO (2 mg/m^3^) and SO_2_ (44 µg/m^3^). The other two gaseous species, NO_2_ and O_3_, on the contrary, demonstrate great urban differences. Large NO_2_ concentrations are mostly distributed in the Suzhou-Wuxi-Changzhou Metropolitan region and nearby areas as a result of the more local vehicular exhaust emissions. Throughout the year, O_3_ is one of the scarcest atmospheric pollutants in the winter under the conditions of low temperatures, short sunshine duration, and weak solar radiation intensity. Ozone pollution for Zigong and Nanchong (~80 µg/m^3^) is more dependent on traffic vehicle emissions inside the Sichuan Basin [17].

**Figure 3:**
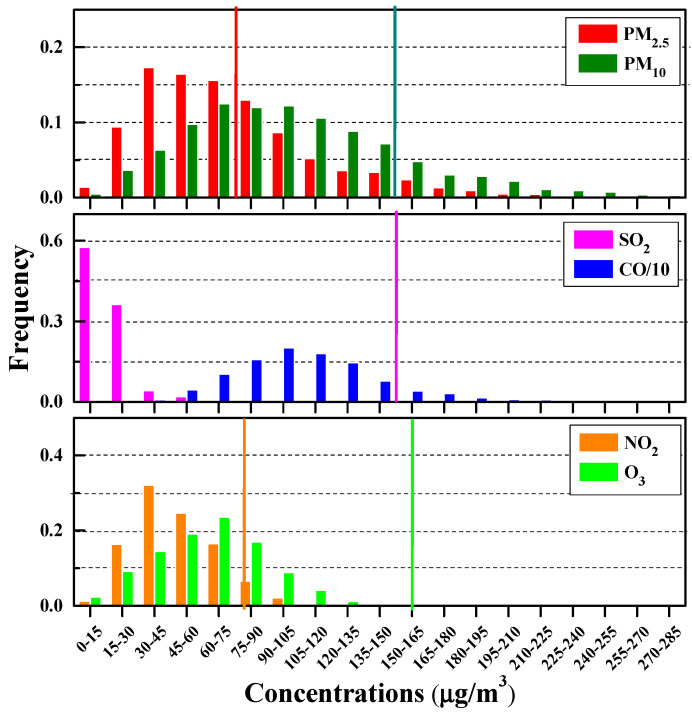
Frequency distribution of six criteria pollutants during the wintertime in the YREB. The perpendicular lines represent the Grade II standard for the ambient criteria air pollutants, except CO.

Figure 3 exhibits the frequency distributions of daily six ambient air pollutants. The average mass concentrations of PM_2.5_ and PM_10_ are 73 and 104 µg/m^3^. PM_2.5_ (PM_10_) concentrations in the range of 30–90 (45–135) µg/m^3^ dominate most days, accounting for about 62% (66%). About 83% and 40% of the PM_2.5_ are above the corresponding CAAQS daily Grade I (35 µg/m^3^) and Grade II (75 µg/m^3^) standards for PM_2.5_ and PM_2.5_, and 86% and 16% of the PM_10_ exceed the CAAQS Grade I (50 µg/m^3^) and Grade II standards for PM_10_ (150 µg/m^3^), respectively. The O_3_ concentrations average as 62 µg/m^3^, with nearly 8% exceeding the CAAQS-I standard (100 µg/m^3^). Similarly, the SO_2_ and CO averages are 16 µg/m^3^ and 1.1 mg/m^3^, respectively, with a tiny percentage (~1%) above the Grade I daily standards (50 µg/m^3^ for SO_2_ and 2 mg/m^3^ for CO). NO_2_ is the major gaseous pollutant in the wintertime. The mean NO_2_ concentration is 47 µg/m^3^, with a range of 10–110 µg/m^3^. The frequency distributions of daily NO_2_ indicate that 59% and 6% NO_2_ could not meet the Grade I (40 µg/m^3^) and Grade II (80 µg/m^3^) standards, respectively.

**Figure 4:**
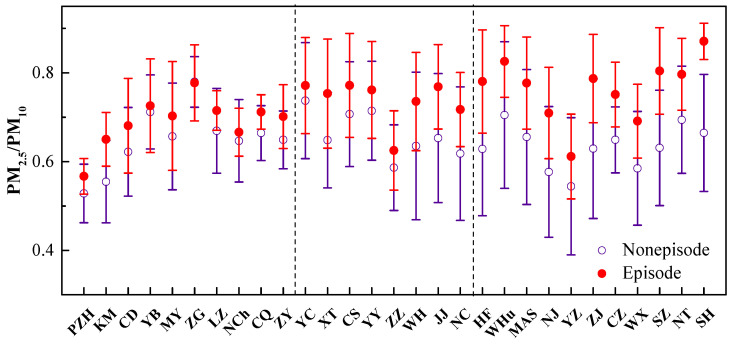
Mean PM_2.5_/PM_10_ ratios and standard deviations in the Episode and Nonepisode days.

The formation mechanisms and sources of PM_2.5_ are complicated, and recent research has stated that secondary aerosol components probably play an important role in the PM_2.5_ mass [22,35]. Besides, the primary sources might be a combination of resuspended dust, biomass combustion, transportation, and industrial activities, etc. The PM_2.5_-to-PM_10_ ratios during both the episode (daily average PM_2.5_ > 75 µg/m^3^) and nonepisode days are presented in Figure 4. The mean PM_2.5_/PM_10_ ratios increase from 0.65 on nonepisode days to 0.69 on episode days in the upstream cities, and the corresponding values are 0.66 vs. 0.74 and 0.63 vs. 0.76 for the midstream and downstream cities, respectively. The remarkably increased PM_2.5_/PM_10_ levels on episode days highlights that PM_2.5_ accounts for a dominant fraction of the PM_10_ mass, and the contribution of secondary aerosols to the PM_2.5_ mass significantly increases. Moreover, larger differences of the ratios between episode and nonepisode days are exposed downstream (0.13) as compared to upstream (0.04) and midstream (0.08). Panzhihua, Zhuzhou, and Yangzhou have relatively lower PM_2.5_/PM_10_ ratios, implying more primary PM sources may occur in those three cities. The stable atmospheric conditions in the wintertime are conducive to the secondary PM accumulation and coarse particles dry deposition, which eventually result in the dominance of fine particles in PM_10_. The combined control strategy, including anthropogenic primary emissions and the secondary PM formation, can alleviate the ambient pollution more effectively.

### 3.2. Spatial Variability

**Figure 5:**
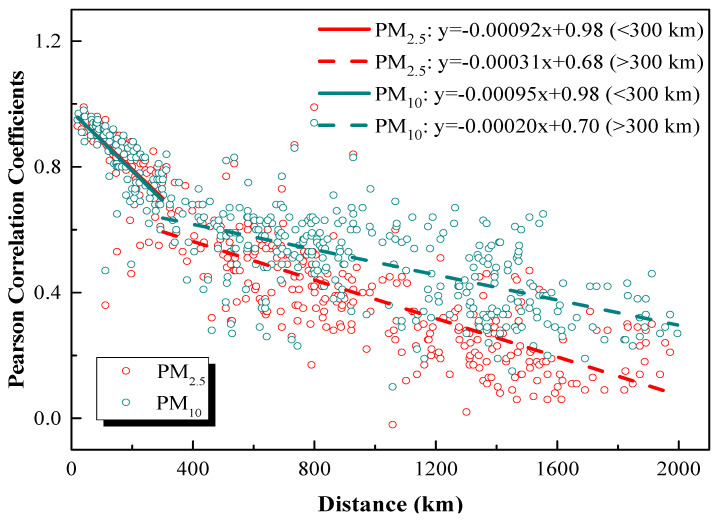
Pearson correlation coefficients of PM_2.5_ and PM_10_ between cities over the YREB.

The regional relationship of daily average PM_2.5_ and PM_10_ between all studied cities is investigated using the Pearson correlation coefficient (*r*) analysis. The corresponding results are demonstrated in Table 3, ranging from 0 to 0.3 (weak correlation), 0.3 to 0.7 (moderate correlation), and 0.7 to 1.0 (strong correlation). The correlation coefficients are further compared in light of the distance between cities (Figure 5). The city pairs located closer to each other present with better correlations. Given in bold in Table 3, most city pairs in the downstream of the Yangtze River show strong correlations (distance < 285 km), except Hefei with Wuxi, Suzhou, Nantong, and Shanghai (290 km < distance < 410 km). Basically, cities in the downstream moderately correlate with those in the midstream (480 km < distance < 980 km) while weakly correlating with those in the upstream (1050 km < distance < 2000 km). The correlation coefficients for PM_2.5_ are generally lower as compared to PM_10_. It may be caused by the following reasons, such as local primary emission sources and meteorological, geographic, and geological conditions [40]. When the distance between cities exceeds 300 km, the *r* values of PM_2.5_ and PM_10_ seldom outstrip 0.7, indicating that, besides abating the local emissions, collaborative controls across administrative borders are needed for any urban areas.

### 3.3. Major Pollutant

**Figure 6:**
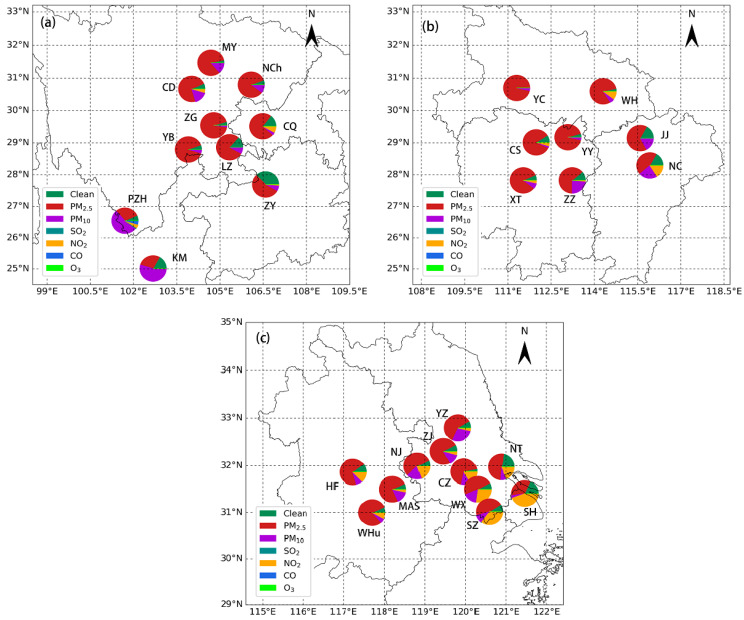
The proportion of major pollutants for the studied cities over the YREB (**a**) upstream, (**b**) midstream, and (**c**) downstream.

Figure 6 shows the fractions of major pollutants for cities in the upstream (a), midstream (b), and downstream (c) regions. One of the six criteria pollutants with the largest AQI as AQI > 50 is defined as the major pollutant. 

PM_10_ is the dominant pollutant affecting Kunming and Panzhihua, occupying more than half of all pollutants, possibly caused more by the influence of local dust discharge. For other cities in the upstream region, PM_2.5_ dominates over PM_10_ as the most significant air contaminant with a frequency of 55–93%, followed by PM_10_ (2–16%) and NO_2_ (0–8%). SO_2_ rarely acts as the primary pollutant except 1% for Panzhihua, and other gas species, including CO and O_3_, are never found as major pollutants. PM_2.5_ is still the main pollutants in the midstream region, and PM_10_ is still the second-most frequent pollutant for most cities, whereas NO_2_ is the second-most frequent pollutant for Changsha and Wuhan. Curiously, NO_2_ pollution is relatively severe in the downstream region and appears to be the most important pollutant for Shanghai (44%) and the second-most important pollutant for Suzhou (35%), Wuxi (27%), Nanjing (19%), Nantong (18%), Changzhou (16%), Hefei (13%), and Wuhu (8%). NO_2_ emissions mainly come from traffic vehicles, power stations, and industries with a huge consumption of fuels—among which, motor vehicles account for the largest percentage. As shown in Table 1, the expansion of vehicular counts is observed downstream. For example, the total number of vehicles reached 3.6, 3.6, 2.6, 1.9, 1.8, and 1.2 million for Shanghai, Suzhou, Nanjing, Nantong, Wuxi, and Changzhou, respectively. The rapid growth of traffic vehicles and the resulting exhaust emission lead to a sharp increase of NOx and volatile organic compounds, which has a great impact on the air quality. Since NO_2_ is the precursor of O_3_ and PM, the strict NO_x_ emission control measures should be modified in the near feature. In addition, due to the strong chemical activity and short lifetime of NO_2_, its concentration is less affected by regional transport. Like the upstream region, no O_3_, CO, and SO_2_ pollutions are found in the midstream and downstream regions. In brief, air pollution in the YREB has evident spatial variability, and proper collaborative control measures of PM and NO_x_ should be taken to effectively abate the air quality of different cities in the winter.

### 3.4. Health Risks Assessment

Based on the daily average values of pollutants, three approaches, including the AQI, AAQI, and HAQI indices, are calculated to characterize the health risks for individual cities.

**Figure 7:**
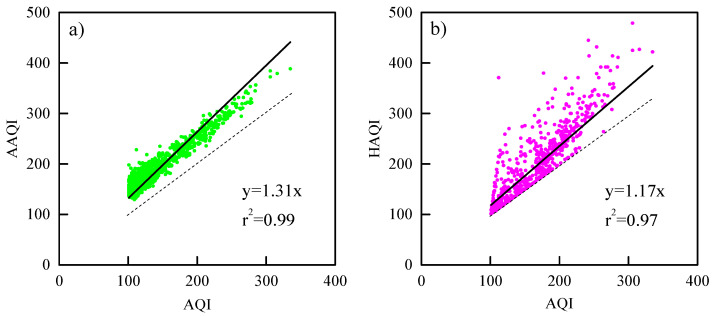

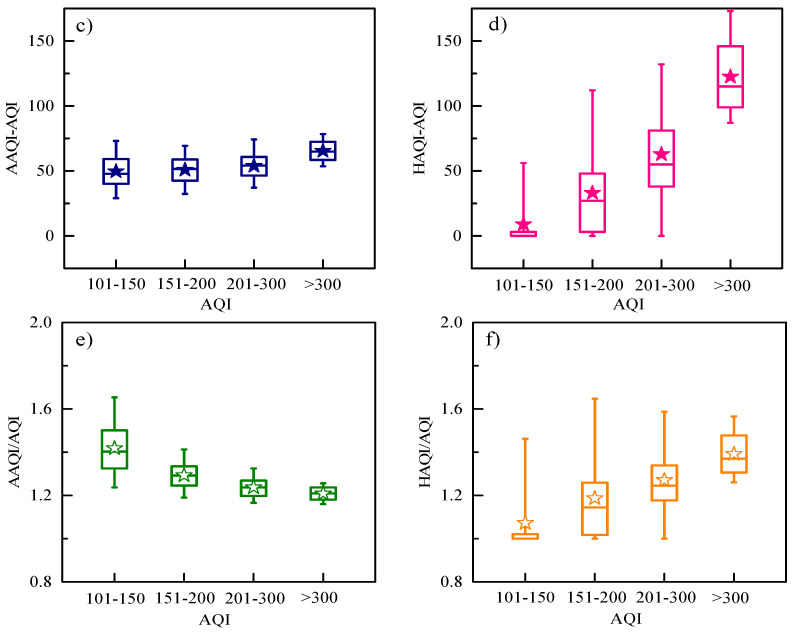
Scatter plots between the air quality index (AQI) and (**a**) aggregate AQI (AAQI) and (**b**) health risk-based AQI (HAQI). Whiskers box plots between the AQI and (**c**) AAQI–AQI, (**d**) HAQI–AQI, (**e**) AAQI/AQI, and (**f**) HAQI/AQI. The top and bottom whiskers show the 95th and 5th percentiles, the upper and lower boundaries of the central box indicate the 75th and 25th percentiles, the middle line of the box indicates the median, and the pentacle indicates the arithmetic average.

In the case of AQI < 100 and AQI = HAQI, the air has no obvious impact on human health. Thus, it is defined as a healthy day; otherwise, it is defined as an unhealthy day when AQI > 100. As shown by the scattered plots with the comparisons of three calculated indices for all cities in Figure 7a,b, both AAQI and HAQI values are greater than the corresponding AQI on unhealthy days, suggesting that the AQI obtained based on the maximum concentrations of the six criteria pollutants seriously underestimate the air pollution level, yet the AAQI and HAQI show higher health risks due to their combined consideration of the impact of multi-pollutants on health. Seen as a whole, the average values of the three indices follow the order of AAQI (199) > HAQI (170) > AQI (148) when AQI > 100. The correlation coefficients (*r^2^*) are 0.99 and 0.97 for AAQI vs. AQI and HAQI vs. AQI, and the slopes of zero-crossing linear fitting are 1.31 and 1.17, respectively. Moreover, the data are divided into four AQI-based health risk categories (101–150 light pollution, 151–200 moderate pollution, 201–300 serious pollution, and > 300 very severe pollution). As shown in Figure 7c–f, on the light pollution and moderate pollution days, both the estimated AAQI–AQI gaps and AAQI/AQI ratios are obviously greater than the HAQI–AQI gaps and HAQI/AQI ratios. The averaged values are 49.9 vs. 9.4 (gap) and 1.4 vs. 1.1 (ratio) for light pollution and 52.0 vs. 33.1 (gap) and 1.3 vs. 1.2 (ratio) for moderate pollution. However, the HAQI gaps and ratios become smartly higher than the AAQI on serious pollution days (63.3 vs. 54.5 and 1.3 vs. 1.2) and very severe pollution days (123.7 vs. 66.6 and 1.4 vs. 1.2), suggesting the AAQI approach likely under-reports the health risks on days when the AQI > 200.

**Figure 8:**
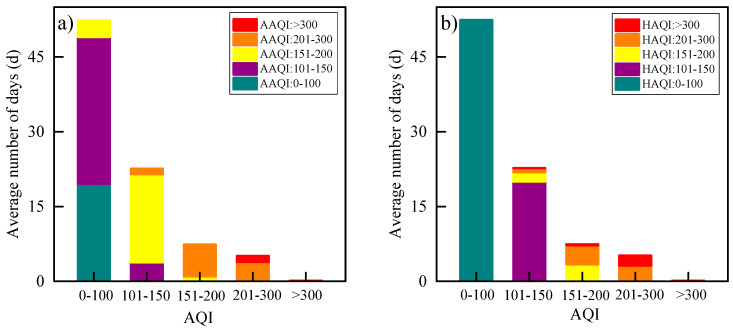
Comparisons of AQI-classified health risk categories with the (**a**) AAQI and (**b**) HAQI classifications (averaged data from 29 cities).

Figure 8 depicts the number of days of five health risk categories on account of the AQI during studied period (average of all cities). Each category is then reclassified according to distinct AAQI and HAQI levels. For AQI-based healthy days (AQI < 100 and AQI = HAQI), 63% of days are light or moderate pollution if based on the AAQI. For the AQI-based risk category of light pollution, 78% (8%) and 5% (3%) of the days are moderate pollution and serious pollution if based on the AAQI (HAQI). For AQI-based moderate pollution, the AAQI and HAQI classify 86% and 54% of days into serious pollution, respectively. The AQI-based serious pollution days account for a few days, yet there is still 24% of very severe pollution days based on the AAQI, and the ratio is up to 39% based on the HAQI. The results show that the counted days obtained by different approaches used to consider the health risks are inconsistent. The AAQI and HAQI-based health risks in many cases are clearly greater than what the government AQI propose, especially for the days when the AQI >100, indicating that the impact of the actual air quality on human health is more serious.

**Figure 9:**
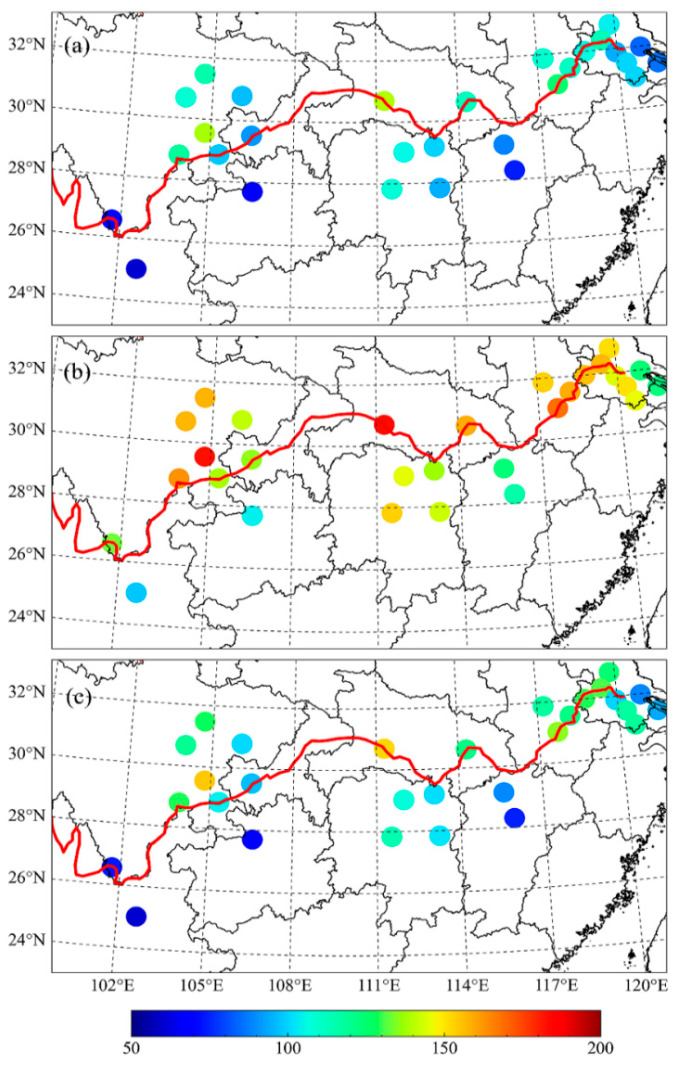
Wintertime average values of the (**a**) AQI, (**b**) AAQI, and (**c**) HAQI.

Figure 9 illustrates the spatial distribution of the mean AQI, AAQI, and HAQI values in the wintertime. Air pollution appears to be more serious on the north bank of the Yangtze River. Based on the AQI, among all cities, only 14 cities are under light pollution, and no city is found to be under moderate pollution. While based on the HAQI, the number of cities with light pollution and moderate pollution are 17 and 2, and the numbers are 15 and 13 based on the AAQI, respectively. The wintertime air quality in most studied cities over the YREB is still not optimistic.

**Figure 10:**
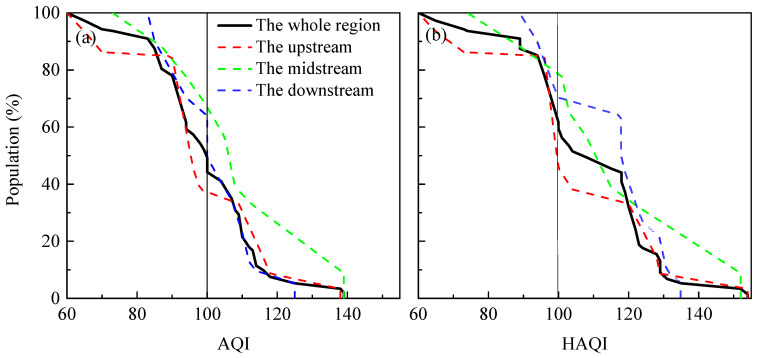
The cumulative population distribution (in % of the corresponding regions) based on the wintertime average (**a**) AQI and (**b**) HAQI.

To visually assess the proportion of humans exposed to air pollution, the cumulative population distribution against the mean HAQI during wintertime is calculated, and the results are displayed in Figure 10b. On average, the proportions of people exposed to air pollution in the YREB are approximately 63% (HAQI > 100). Different spatial scales have diverse cumulative distributions of population weights based on individual HAQI values. Nearly 50% of people are faced with polluted air for the upstream. By contrast, higher concentrations of PM and NO_x_ in the midstream and downstream pose higher risks of exposure to the local population; and the proportions of people exposed to air pollution are 70% and 80% for the midstream and downstream, respectively. The discrepancy between Figure 10a and Figure 10b once more gives prominence to the advantage of the HAQI that can better reflect the exposure–response relationships of air pollutants to human health. At present, more valid measures should be taken to lighten the wintertime air pollution in the YREB.

## 4. Conclusions

In this work, we utilized the hourly monitoring data of six criteria pollutants (PM_2.5_, PM_10_, SO_2_, CO, NO_2_, and O_3_) to discuss the air pollution characteristics and associated human health risks in 29 typical cities over the YREB during December 2017–February 2018. From the spatial distribution of the PM, cities on the north bank of the middle and lower reaches were more polluted than those on the south bank of the upper reaches of the Yangtze River. The Pearson correlation coefficient of the PM seldom outstripped 0.7 if the distance between the city pairs was over 300 km. The PM_2.5_/PM_10_ ratios highlighted that the contribution of secondary aerosols to the PM_2.5_ mass increased on episode days (PM_2.5_ > 75 μg/m^3^). Compared to CO and SO_2_, NO_2_ and O_3_ displayed more obvious intercity differences. High NO_2_ concentrations occurred in the Suzhou-Wuxi-Changzhou Metropolitan region and nearby areas due to the more local vehicular exhaust discharge. PM_2.5_, PM_10_, and NO_2_ were the three most frequent major pollutants in the wintertime. The AQI, AAQI, and HAQI approaches were further used to characterize the spatial distribution of the air pollution. The HAQI approach took into account the population and reflected the exposure–response relationship of air pollutants to human health, which can better assess the human health risks of air pollution. During the study period, approximately 50%, 70%, and 80% of people in the upstream, midstream, and downstream of the YREB were exposed to air pollution (HAQI >100). More efficient pollution control strategies should be adopted to improve the air quality and reduce health risks by further strengthening interregional cooperation.

## Figures and Tables

**Table 1:** Basic information of 29 cities over the Yangtze River Economic Belt (YREB) by the end of 2017. GDP: gross domestic product.

Cities	Latitude (Degree)	Longitude (Degree)	Area (Km^2^)	Population (Million)	GDP (Billion)	Vehicle (Ten Thousand)	Number of Monitoring Sites
**The upstream**							
Panzhihua (PZH)	26.6	101.7	7401	1.2	114.4	26.0	5
Kunming (KM)	25.0	102.7	21,281	5.7	485.8	213.5	7
Chengdu (CD)	30.7	104.0	14,335	16.3	1388.9	398.2	10
Yibin (YB)	28.8	104.6	13,271	4.6	184.7	32.3	6
Mianyang (MY)	31.5	104.7	20,248	4.9	207.5	17.5	4
Zigong (ZG)	29.4	104.8	4381	2.9	131.2	22.4	6
Luzhou (LZ)	28.9	105.4	12,232	4.3	159.6	37.8	4
Nanchong (NCh)	30.8	106.1	12,477	6.4	182.8	46.2	6
Chongqing (CQ)	29.5	106.5	82,400	34.0	1950.0	320.7	21
Zunyi (ZY)	27.7	106.9	30,762	6.2	272.7	70.1	5
**The midstream**							
Yichang (YC)	30.7	111.3	21,230	4.1	385.7	56.7	5
Xiangtan (XT)	27.8	112.9	5006	2.9	205.6	61.1	7
Changsha (CS)	28.1	113.0	11,816	8.2	1053.5	256.5	10
Yueyang (YY)	29.4	113.1	14,858	5.8	325.8	49.0	6
Zhuzhou (ZZ)	27.8	113.1	11,248	4.0	252.2	44.0	7
Wuhan (WH)	30.6	114.3	8569	11.1	1341.0	261.0	10
Jiujiang (JJ)	29.5	115.6	19,085	4.9	241.4	65.9	8
Nanchang (NC)	28.6	115.9	7402	5.5	500.3	97.0	10
**The downstream**							
Hefei (HF)	31.8	117.2	11,445	7.6	721.4	169.7	10
Wuhu (WHu)	31.3	118.4	6026	3.9	306.6	47.7	4
Maanshan (MAS)	31.7	118.5	4049	2.3	173.8	31.1	5
Nanjing (NJ)	32.0	118.8	6587	8.4	1171.5	257.9	9
Yangzhou (YZ)	32.4	119.4	6591	4.5	506.5	94.0	5
Zhenjiang (ZJ)	32.2	119.5	3840	3.2	410.5	49.6	5
Changzhou (CZ)	31.8	120.0	4374	4.7	662.2	122.8	9
Wuxi (WX)	31.6	120.3	4627	6.6	1051.2	176.5	8
Suzhou (SZ)	31.3	120.6	8657	10.7	1730.0	355.7	8
Nantong (NT)	32.0	120.9	10,549	7.3	773.5	187.3	5
Shanghai (SH)	31.4	121.5	6341	14.6	3013.4	361.0	10

**Table 2:** Wintertime average concentrations and associated standard deviations of six criteria pollutants in all cities. PM: particle matter and CAAQS: Chinese Ambient Air Quality Standard.

Cities	PM_2.5_ (μg/m^3^)	PM_10_ (μg/m^3^)	SO_2_ (μg/m^3^)	CO (mg/m^3^)	NO_2_ (μg/m^3^)	O_3_ 8 h (μg/m^3^)
PZH	45.7 ± 14.7	86.7 ± 27.1	44.4 ± 16.6	2.2 ± 0.7	47.2 ± 12.1	70.0 ± 21.6
KM	37.6 ± 15.8	66.2 ± 22.0	14.5 ± 4.1	1.0 ± 0.2	34.4 ± 7.8	76.3 ± 27.4
CD	79.5 ± 36.1	123.6 ± 55.8	10.9 ± 3.5	1.1 ± 0.3	52.1 ± 16.6	71.7 ± 23.6
YB	87.8 ± 40.2	123.7 ± 56.0	21. 9± 5.9	1.3 ± 0.3	45.1 ± 11.2	54.6 ± 29.8
MY	84.6 ± 41.4	128.0 ± 69.4	7.8 ± 3.2	1.0 ± 0.3	40.3 ± 14.9	68.0 ± 23.5
ZG	105.4 ± 45.3	136.4 ± 58.2	19.2 ± 6.7	1.2 ± 0.3	44.4 ± 16.4	79.7 ± 35.1
LZ	71.4 ± 37.3	104.7 ± 52.9	17.0 ± 7.2	0.8 ± 0.3	41.5 ± 13.8	42.3 ± 27.6
NCh	71.3 ± 28.2	110.5 ± 43.5	10.4 ± 3.6	1.0 ± 0.2	39.9 ± 14.4	79.7 ± 24.6
CQ	65.3 ± 35.1	96.5 ± 49.8	11.1 ± 3.5	1.1 ± 0.2	50.9 ± 14.2	35.2 ± 20.3
ZY	45.9 ± 24.8	69.9 ± 36.2	23.0 ± 11.9	1.0 ± 0.2	33.6 ± 12.6	59.4 ± 23.3
YC	105. 7± 39.0	137.7 ± 49.4	13.5 ± 2.4	1.4 ± 0.2	45.9 ± 13.6	56.8 ± 23.6
XT	80.6 ± 38.8	111.6 ± 48.5	20.1 ± 12.9	1.0 ± 0.2	45.8 ± 18.3	65.7 ± 29.2
CS	78.3 ± 37.8	92.2 ± 43.8	13.2 ± 8.6	1.0 ± 0.2	45.8 ± 18.6	60.5 ± 28.2
YY	74.3 ± 26.0	101.8 ± 34.1	9.9 ± 4.7	1.1 ± 0.2	30.7 ± 12.5	67.5 ± 26.7
ZZ	68.4 ± 33.4	114.5 ± 51.4	21.4 ± 10.5	0.9 ± 0.2	44.9 ± 16.0	60.5 ± 29.6
WH	83.3 ± 35.1	106.7 ± 42.3	12.2 ± 7.0	1.2 ± 0.3	56.4 ± 22.0	54.4 ± 25.2
JJ	63.9 ± 30.9	86.1 ± 40.4	15.5 ± 7.1	0.8 ± 0.2	35.4 ± 13.2	63.3 ± 20.5
NC	49.5 ± 22.7	82.5 ± 36.7	12.6 ± 7.1	1.1 ± 0.3	44.3 ± 15.9	63.1 ± 26.7
HF	81.1 ± 43.0	100.5 ± 45.4	10.2 ± 5.8	1.1 ± 0.3	53.6 ± 22.2	63.3 ± 22.5
WHu	94.1 ± 54.1	105.1 ± 52.3	16.1 ± 6.8	1.1 ± 0.4	57.0 ± 20.4	60.5 ± 26.3
MAS	82.6 ± 48.3	113.0 ± 54.6	23.4 ± 9.3	1.2 ± 0.4	51.3 ± 20.1	63.1 ± 21.4
NJ	75.7 ± 51.1	118.3 ± 64.7	14.7 ± 5.3	1.0 ± 0.4	58.4 ± 23.2	61.3 ± 21.1
YZ	73.7 ± 41.1	129.1 ± 66.1	13.4 ± 7.0	1.0 ± 0.4	46.3 ± 23.4	59.9 ± 19.7
ZJ	84.5 ± 53.3	110.0 ± 59.8	14.3 ± 8.1	0.8 ± 0.4	52.3 ± 25.5	60.9 ± 20.1
CZ	68.6 ± 34.8	98.7 ± 40.9	18.4 ± 4.5	1.3 ± 0.3	56.2 ± 15.8	44.4 ± 13.5
WX	69.9 ± 46.9	111.0 ± 62.1	15.2 ± 6.1	1.4 ± 0.4	57.3 ± 22.2	60.2 ± 21.7
SZ	67.9 ± 46.2	96.5 ± 53.1	15.0 ± 6.6	1.0 ± 0.4	61.7 ± 24.7	61.6 ± 21.0
NT	58.8 ± 38.3	78.9 ± 45.4	19.0 ± 6.9	1.0 ± 0.3	45.0 ± 21.5	73.0 ± 17.7
SH	52.9 ± 36.7	69.2 ± 38.3	13.7 ± 5.1	0.9 ± 0.3	56.8 ± 21.9	73.1 ± 19.3
CAAQS-I/II	35/75	50/150	50/150	2/4	40/80	100/160

**Table 3:** Pearson correlation coefficients (*r*) for PM_2.5_ (cells above the diagonal) and PM_10_ (cells below the diagonal) between studied cities. 0 < *r* ≤ 0.3 (weak correlation), 0.3 < *r* ≤ 0.7 (moderate correlation), and 0.7 < *r* ≤ 1.0 (strong correlation, in bold).

	PZH	KM	CD	YB	MY	ZG	LZ	NCh	CQ	ZY	YC	XT	CS	YY	ZZ	WH	JJ	NC	HF	WHu	MAS	NJ	YZ	ZJ	CZ	WX	SZ	NT	SH
**PZH**	—	0.46	0.48	0.50	0.38	0.45	0.45	0.38	0.38	0.31	0.27	0.24	0.23	0.17	0.29	0.17	0.23	0.27	0.25	0.34	0.28	0.28	0.29	0.30	0.29	0.28	0.30	0.24	0.27
**KM**	0.49	—	0.34	0.32	0.31	0.31	0.35	0.26	0.37	0.51	0.00	0.23	0.21	0.14	0.29	0.02	0.11	0.13	0.06	0.09	0.13	0.09	0.15	0.09	0.11	0.14	0.18	0.14	0.21
**CD**	0.43	0.26	—	**0.82**	**0.92**	**0.86**	**0.82**	**0.88**	**0.85**	0.62	0.62	0.40	0.36	0.30	0.48	0.34	0.34	0.41	0.34	0.33	0.29	0.28	0.37	0.35	0.22	0.18	0.19	0.14	0.11
**YB**	0.44	0.28	**0.86**	—	**0.73**	**0.89**	**0.94**	**0.75**	**0.80**	0.56	0.59	0.41	0.37	0.32	0.46	0.40	0.43	0.43	0.32	0.33	0.27	0.26	0.30	0.31	0.21	0.17	0.18	0.10	0.11
**MY**	0.29	0.23	**0.92**	**0.78**	—	**0.81**	**0.75**	**0.84**	**0.78**	0.63	0.57	0.37	0.35	0.29	0.45	0.30	0.31	0.34	0.29	0.28	0.27	0.25	0.32	0.31	0.20	0.14	0.16	0.12	0.08
**ZG**	0.41	0.30	**0.88**	**0.91**	**0.83**	—	**0.90**	**0.85**	**0.82**	0.64	0.60	0.47	0.44	0.35	0.54	0.41	0.45	0.45	0.32	0.33	0.30	0.28	0.33	0.33	0.23	0.18	0.18	0.14	0.12
**LZ**	0.44	0.37	**0.83**	**0.91**	**0.73**	**0.90**	—	**0.76**	**0.88**	0.63	0.61	0.45	0.41	0.36	0.50	0.39	0.42	0.44	0.27	0.29	0.23	0.21	0.29	0.27	0.18	0.13	0.14	0.07	0.06
**NCh**	0.35	0.25	**0.87**	**0.74**	**0.80**	**0.85**	**0.78**	—	**0.83**	0.66	0.58	0.39	0.36	0.29	0.49	0.30	0.37	0.40	0.25	0.25	0.22	0.19	0.28	0.25	0.17	0.12	0.13	0.08	0.06
**CQ**	0.41	0.35	**0.76**	**0.73**	0.66	**0.80**	**0.88**	**0.81**	—	0.68	0.55	0.45	0.40	0.33	0.51	0.35	0.38	0.44	0.28	0.30	0.25	0.21	0.35	0.28	0.20	0.16	0.18	0.10	0.09
**ZY**	0.27	0.45	0.68	0.62	0.62	0.68	0.69	**0.75**	0.70	—	0.37	0.46	0.42	0.34	0.60	0.17	0.37	0.46	0.12	0.20	0.20	0.14	0.24	0.19	0.20	0.18	0.22	0.19	0.21
**YC**	0.36	0.10	0.67	0.60	0.58	0.63	0.63	0.65	0.54	0.49	—	0.58	0.55	0.55	0.53	0.55	0.56	0.52	0.53	0.48	0.43	0.41	0.42	0.46	0.34	0.28	0.29	0.28	0.22
**XT**	0.33	0.32	0.65	0.62	0.60	0.64	0.61	0.63	0.55	0.62	**0.71**	—	**0.99**	**0.87**	**0.93**	**0.75**	**0.77**	**0.81**	0.61	0.54	0.55	0.53	0.52	0.53	0.47	0.42	0.43	0.39	0.36
**CS**	0.31	0.29	0.60	0.56	0.56	0.59	0.55	0.60	0.49	0.60	0.63	**0.94**	—	**0.89**	**0.92**	**0.77**	**0.76**	**0.78**	0.62	0.55	0.55	0.52	0.52	0.53	0.47	0.42	0.43	0.39	0.36
**YY**	0.19	0.22	0.53	0.51	0.48	0.49	0.48	0.49	0.37	0.48	0.69	**0.86**	**0.81**	—	**0.84**	**0.79**	**0.79**	**0.73**	0.62	0.61	0.60	0.57	0.52	0.54	0.49	0.45	0.45	0.40	0.38
**ZZ**	0.33	0.31	0.60	0.57	0.53	0.63	0.56	0.62	0.53	0.60	0.65	**0.95**	**0.91**	**0.84**	—	0.65	**0.78**	**0.78**	0.52	0.54	0.54	0.52	0.53	0.53	0.49	0.44	0.44	0.42	0.38
**WH**	0.42	0.27	0.73	0.67	0.67	0.65	0.62	0.64	0.57	0.58	0.68	**0.75**	**0.71**	**0.71**	**0.70**	—	**0.83**	**0.70**	**0.80**	0.66	0.64	0.61	0.53	0.56	0.47	0.43	0.41	0.34	0.34
**JJ**	0.34	0.29	0.64	0.62	0.61	0.58	0.59	0.62	0.54	0.66	0.64	**0.82**	**0.76**	**0.74**	**0.76**	**0.80**	—	**0.84**	**0.80**	0.75	0.74	0.68	0.57	0.62	0.61	0.59	0.59	0.51	0.53
**NC**	0.33	0.24	0.61	0.57	0.54	0.56	0.56	0.61	0.51	0.63	0.65	**0.83**	**0.78**	**0.77**	**0.81**	**0.74**	0.83	—	0.60	0.56	0.54	0.51	0.51	0.49	0.49	0.47	0.47	0.42	0.44
**HF**	0.43	0.28	0.67	0.62	0.64	0.59	0.54	0.57	0.49	0.47	0.64	**0.71**	0.64	0.63	0.64	**0.84**	0.79	0.69	—	**0.85**	**0.86**	**0.81**	**0.73**	**0.76**	0.70	0.68	0.67	0.59	0.58
**WHu**	0.42	0.19	0.49	0.50	0.40	0.48	0.43	0.41	0.34	0.32	0.56	0.64	0.58	0.67	0.62	0.68	0.56	0.56	**0.77**	—	**0.97**	**0.94**	**0.84**	**0.90**	**0.90**	**0.87**	**0.85**	**0.78**	**0.78**
**MAS**	0.42	0.29	0.55	0.54	0.51	0.51	0.44	0.46	0.38	0.39	0.54	0.68	0.62	0.66	0.68	**0.73**	0.69	0.63	**0.85**	**0.88**	—	**0.97**	**0.87**	**0.93**	**0.92**	**0.89**	**0.87**	**0.81**	**0.80**
**NJ**	0.42	0.25	0.56	0.56	0.52	0.51	0.45	0.42	0.37	0.33	0.51	0.65	0.58	0.63	0.64	0.69	0.64	0.57	**0.80**	**0.87**	**0.96**	—	**0.88**	**0.96**	**0.93**	**0.89**	**0.86**	**0.82**	**0.79**
**YZ**	0.39	0.25	0.52	0.50	0.45	0.47	0.43	0.42	0.39	0.37	0.52	0.63	0.58	0.59	0.62	0.64	0.60	0.59	**0.83**	**0.85**	**0.91**	**0.94**	—	**0.95**	**0.88**	**0.82**	**0.80**	**0.78**	**0.71**
**ZJ**	0.43	0.25	0.55	0.53	0.48	0.49	0.46	0.42	0.42	0.36	0.53	0.64	0.57	0.58	0.63	0.67	0.61	0.59	**0.75**	**0.85**	**0.92**	**0.96**	**0.97**	—	**0.94**	**0.88**	**0.84**	**0.84**	**0.77**
**CZ**	0.38	0.23	0.42	0.43	0.38	0.40	0.35	0.35	0.33	0.31	0.42	0.57	0.53	0.53	0.59	0.57	0.58	0.53	0.70	**0.80**	**0.92**	**0.93**	**0.95**	**0.95**	—	**0.91**	**0.95**	**0.93**	**0.90**
**WX**	0.38	0.26	0.39	0.39	0.34	0.35	0.29	0.33	0.29	0.32	0.38	0.54	0.47	0.52	0.56	0.55	0.58	0.54	0.68	**0.76**	**0.90**	**0.89**	**0.90**	**0.90**	**0.96**	—	**0.99**	**0.93**	**0.95**
**SZ**	0.46	0.29	0.40	0.39	0.37	0.34	0.29	0.33	0.28	0.35	0.39	0.54	0.47	0.51	0.55	0.54	0.59	0.54	0.68	**0.72**	**0.88**	**0.86**	**0.88**	**0.87**	**0.93**	**0.98**	—	**0.94**	**0.96**
**NT**	0.29	0.27	0.36	0.34	0.32	0.30	0.23	0.27	0.19	0.29	0.34	0.49	0.44	0.46	0.51	0.48	0.54	0.49	0.60	0.66	**0.84**	**0.83**	**0.87**	**0.85**	**0.92**	**0.93**	**0.94**	—	**0.95**
**SH**	0.27	0.33	0.32	0.29	0.29	0.26	0.20	0.28	0.15	0.37	0.33	0.47	0.41	0.47	0.49	0.44	0.54	0.51	0.53	0.60	**0.76**	**0.72**	**0.75**	**0.73**	**0.81**	**0.88**	**0.91**	**0.94**	—

## Supplementary Materials

The following are available online at https://www.mdpi.com/1660-4601/17/24/9172/s1, Table S1: Concentration limits for AQI calculation.

## References

[1] Cao J., Xu H., Xu Q., Chen B., Kan H. (2012). Fine particulate matter constituents and cardiopulmonary mortality in a heavily polluted Chinese city. Environ. Health Perspect..

[2] Shang Y., Sun Z., Cao J., Wang X., Zhong L., Bi X., Li H., Liu W., Zhu T., Huang W. (2013). Systematic review of Chinese studies of short-term exposure to air pollution and daily mortality. Environ. Int..

[3] Fang D., Chen B., Hubacek K., Ni R., Chen L., Feng K., Lin J. (2019). Clean air for some: Unintended spillover effets of regional air pollution policies. Sci. Adv..

[4] Zhang X., Mao M., Yin Y., Tang S. (2020). The absorption Ångstrom exponent of black carbon with brown coatings: Effects of aerosol microphysics and parameterization. Atmos. Chem. Phys..

[5] Li R., Wang Z., Cui L., Fu H., Zhang L., Kong L., Chen W., Chen J. (2019). Aair pollution characteristics in China during 2015–2016: Spationtemporal variations and key meteorological factors. Sci. Total Environ..

[6] Guo H., Gu X., Ma G., Shi S., Wang W., Zuo X., Zhang X. (2019). Spatial and temporal variation of air quality and six air pollutants in China during 2015–2017. Sci. Rep..

[7] Ma X., Jia H., Sha T., An J., Tian R. (2019). Spatial and seasonal characteristics of particulate matter and gaseous pollution in China: Implications for control policy. Environ. Pollut..

[8] Zeng Y., Cao Y., Qiao X., Seyler B., Tang Y. (2019). Air pollution reduciton in China: Recent success but great challenge for the future. Sci. Total Environ..

[9] Yu F., Wang Q., Yan Q., Jiang N., Wei J., Wei Z., Yin S. (2018). Particle size distribution, cemical composition and meteorological factor analysis: A case study during wintertime snow cover in Zhengzhou, China. Atmos. Res..

[10] Lelieveld J., Evans J.S., Fnais M., Giannadaki D., Pozzer A. (2015). The contribution of outdoor air pollution sources to premature mortality on a global scale. Nature.

[11] Li C., Dai Z., Yang L., Ma Z. (2019). Spatiotemporal Characteristics of Air Quality across Weifang from 2014–2018. Int. J. Environ. Res. Public Health.

[12] Maji K.J., Ye W.F., Arora M., Nagendra S.M.S. (2018). PM2.5-related health and economic loss assessment for 338 Chinese cities. Environ. Inter..

[13] Feng Y., Cheng J., Shen J., Sun H. (2019). Spatial effects of air pollution on public health in China. Environ. Resour. Econ..

[14] Gu H., Cao Y., Elahi E., Jha S.K. (2019). Human health damages related to air polluton in China. Environ. Sci. Pollut. Res..

[15] The Outline of Yangtze River Economic Belt Development ProgramPlan. http://www.gov.cn/xinwen/2016-09/12/content_5107501.htm.

[16] Wang S., Zhou C., Wang Z., Feng K., Hubacek K. (2017). The characteristics and drivers of fine particulate matter (PM2.5) distribution in China. J. Clean. Prod..

[17] Zhao S., Yu Y., Yin D., Qin D., He J., Dong L. (2018). Spatial patterns and temporal varations of six criteria air pollutants during 2015 to 2017 in the city clusters of Sichuan Basin, China. Sci. Total Environ..

[18] Li L., An J.Y., Shi Y.Y., Zhou M., Yan R.S., Huang C., Wang H.L., Lou S.R., Wang Q., Lu Q. (2016). Source apporotionment of surface ozone in the Yangtze River Delta, China in summer of 2013. Atmos. Environ..

[19] Chen T., He J., Lu X., She J., Guan Z. (2016). Spatial and temporal variations of PM_2.5_ and its relation to metorological factor in urban area of Nanjing, China. Int. J. Environ. Res. Public Health.

[20] Nie D., Chen M., Wu Y., Ge X., Hu J., Zhang K., Ge P. (2018). Characterization of fine particulate matter and associated health burden in Nanjing. Int. J. Environ. Res. Public Health.

[21] Zhao Q., Huo J., Yang X., Fu Q., Duan Y., Liu Y., Lin Y., Zhang Q. (2020). Chemical characterization and source identification of submicron aerosols from a year-long real-time observation at a rural site of Shanghai using an Aerosol Chemical Speciation Monitor. Atmos. Res..

[22] Mao M., Zhang X., Yin Y. (2018). Particulate matter and gaseous pollutions in three metropolises along the Chinese Yangtze River: Situation and Implications. Int. J. Environ. Res. Public Health.

[23] Kyrkilis G., Chaloulakou A., Kassomenos P.A. (2007). Development of an aggregate air quality index for an urban mediterranean agglomeration: Relation to potential health effects. Environ. Int..

[24] Wong T.W., San Tam W.W., Yu I.T.S., Lau A.K.H., Pang S.W., Wong A.H. (2013). Developing a risk-based air quality health index. Atmos. Environ..

[25] Hu J., Ying Q., Wang Y., Zhang H. (2015). Characterizing multi-pollutant air pollution in China: Comparison of three air quality indices. Environ. Int..

[26] Shen F., Ge X., Hu J., Nie D., Tian L., Chen M. (2017). Air pollution characteristics and health risks in Henan Province, China. Environ. Res..

[27] Luo H., Guan Q., Lin J., Wang Q., Yang L., Tan Z., Wang N. (2020). Air pollution characteristics and human health risks in key cities of northwest China. J. Environ. Manag..

[28] Shen F., Zhang L., Jiang L., Tang M., Gai X., Chen M., Ge X. (2020). Temporal variations of six ambient criteria air pollutants from 2015 to 2018, their spatial distributions, health risks and relationships with socioeconomic factors during 2018 in China. Environ. Int..

[29] The China National Environmental Monitoring Centre. http://www.cnemc.cn.

[30] The Chinese Statistics Web Links. http://www.tjcn.org/tjgb.

[31] Ministry of Environmental Protection (MEP) (2012). Ambient Air Quality Standards.

[32] Ministry of Environmental Protection (MEP) (2012). Technical Regulation on Ambient Air Quality Index (On Trial).

[33] Swamee P.K., Tyagi A. (1999). Formation of an air pollution index. J. Air Waste Manag. Assoc..

[34] Cairncross E.K., John J., Zunckel M. (2007). A novel air pollution index based on the relative risk of daily mortality associated with short-term exposure to common air pollutants. Atmos. Environ..

[35] He J., Gong S., Yu Y., Yu L., Wu L., Mao H., Song C., Zhao S., Liu H., Li X. (2017). Air pollution characteristics and their relation to meteorological conditions during 2014–2015 in major Chinese cities. Environ. Pollut..

[36] Mao M., Zhang X., Shao Y., Yin Y. (2020). Spatiotemporal Variations and Factors of Air Quality in Urban Central China during 2013–2015. Int. J. Environ. Res. Public Health.

[37] Kuerban M., Waili Y., Fan F., Liu Y., Qin W., Dore A.J., Peng J., Xu W., Zhang F. (2020). Spatio-temporal patterns of air pollution in China from 2015 to 2018 and implications for health risks. Environ. Pollut..

[38] Li C., Liu M., Hu Y., Zhou R., Huang N., Wu W., Liu C. (2020). Spatial distribution characteristics of gaseous pollutants and particulate matter inside a city in the heating season of Northeast China. Sustain. Cities Soc..

[39] Zhao S., Yin D., Yu Y., Kang S., Qin D., Dong L. (2020). PM_2.5_ and O_3_ pollution during 2015-2019 over 367 Chinese cities: Spatiotemporal variations, meteorological and topographical impacts. Environ. Pollut..

[40] Sheppard L., Levy D., Checkoway H. (2001). Correcting for the effects of location and atmospheric conditions on air pollution exposures in a case-crossover study. J. Expos. Anal. Environ. Epidemiol..

