# Supplementary material for: Air Pollution Characteristics and Health Risks in the Yangtze River Economic Belt, China during Winter
Source: Int J Environ Res Public Health. 2020 Dec 8;17(24):9172. doi: 10.3390/ijerph17249172 (PMC7764583; doi:10.3390/ijerph17249172)
Supplement: Supplementary file 1 [file ijerph-17-09172-s001.pdf]

Air pollution characteristics and health risks in the Yangtze River Economic Belt, China during winter

Supplementary data

Mao Mao [1,2,*], Haofei Sun [2] and Xiaolin Zhang [2,*]

[1] School of Binjiang, Nanjing University of Information Science & Technology, Wuxi 214105, China;

[2] Key Laboratory for Aerosol-Cloud-Precipitation of China Meteorological Administration, School of Atmospheric Physics, Nanjing University of Information Science & Technology, Nanjing 210044, China; sunhaofei20@mails.ucas.ac.cn (H.S.)

* Correspondence: mmao@nuist.edu.cn (M.M.); xlnzhang@nuist.edu.cn (X.Z.); Tel.: +86-25-5869-9773

Methods of Air quality index (AQI)

The individual air quality index (AQI_i) for each pollutant ($PM_{2.5}$, PM_{10}, SO_2, CO, NO_2, and O_3) is calculated based on equation (1):

$$AQI_i = \frac{AQI_{i,j} - AQI_{i,j-1}}{(C_{i,j} - C_{i,j-1})} \times \left(C_i - C_{i,j-1}\right) + AQI_{i,j-1}, j > 1,$$

$$AQI_i = AQI_{i,1} \frac{C_i}{C_{i,1}}, \mathrm{j} = 1 \quad (1)$$

Where C_i is the measured daily average concentration of pollutant i. As shown in Table S1, $C_{i,j}$ and $C_{i,j-1}$ are the nearby high and low values of C_i. $AQI_{i,j}$ and $AQI_{i,j-1}$ are the individual air quality indexes for $C_{i,j}$ and $C_{i,j-1}$, respectively. The largest AQI_i value is 500, and the AQI_i value will be set to 500 when the concentration of air pollutant outstrips the highest limit.

After getting each AQI_i, the overall AQI is then calculated by choosing the maximum AQI_i of six pollutants as equation (2)

$$AQI = \max(AQI_1, AQI_2, \ldots\ldots, AQI_n), \mathrm{n} = 1, 2, \ldots\ldots, 6 \quad (2)$$

Table S1. Concentration limits for AQI calculation.

AQI*i*	**$PM_{2.5}$** (μg/m³)	**PM_{10}** (μg/m³)	**SO_2** (μg/m³)	**CO** (mg/m³)	**NO_2** (μg/m³)	**O_3-8h** (μg/m³)
**0**	0	0	0	0	0	0
**50**	35	50	50	2	40	100
**100**	75	150	150	4	80	160
**150**	115	250	475	14	180	215
**200**	150	350	800	24	280	265
**300**	250	420	1600	36	565	800
**400**	350	500	2100	48	750	-
**500**	500	600	2620	60	940	-
